# An Overview of Nrf2 Signaling Pathway and Its Role in Inflammation

**DOI:** 10.3390/molecules25225474

**Published:** 2020-11-23

**Authors:** Sarmistha Saha, Brigitta Buttari, Emiliano Panieri, Elisabetta Profumo, Luciano Saso

**Affiliations:** 1 Department of Cardiovascular, Endocrine-Metabolic Diseases and Aging, Italian National Institute of Health, 00161 Rome, Italy; brigitta.buttari@iss.it (B.B.); elisabetta.profumo@iss.it (E.P.); 2 Department of Physiology and Pharmacology “Vittorio Erspamer”, Sapienza University of Rome, 00161 Rome, Italy; emiliano.panieri@hotmail.it (E.P.); luciano.saso@uniroma1.it (L.S.)

**Keywords:** Nrf2, Keap1, inflammation, oxidative stress, polyphenols

## Abstract

Inflammation is a key driver in many pathological conditions such as allergy, cancer, Alzheimer’s disease, and many others, and the current state of available drugs prompted researchers to explore new therapeutic targets. In this context, accumulating evidence indicates that the transcription factor Nrf2 plays a pivotal role controlling the expression of antioxidant genes that ultimately exert anti-inflammatory functions. Nrf2 and its principal negative regulator, the E3 ligase adaptor Kelch-like ECH- associated protein 1 (Keap1), play a central role in the maintenance of intracellular redox homeostasis and regulation of inflammation. Interestingly, Nrf2 is proved to contribute to the regulation of the heme oxygenase-1 (HO-1) axis, which is a potent anti-inflammatory target. Recent studies showed a connection between the Nrf2/antioxidant response element (ARE) system and the expression of inflammatory mediators, NF-κB pathway and macrophage metabolism. This suggests a new strategy for designing chemical agents as modulators of Nrf2 dependent pathways to target the immune response. Therefore, the present review will examine the relationship between Nrf2 signaling and the inflammation as well as possible approaches for the therapeutic modulation of this pathway.

## 1. Introduction

Inflammation is generally a pervasive response to disturbances in the tissue homeostasis due to a variety of stimuli such as pathogens, tissue injury, or contaminants which involves the activation of innate and adaptive immunity. During the last few decades, inflammation has been the focus of extensive research mainly aimed at elucidating its role in host defense mechanisms; however, at present, inflammation is linked to a wide range of diseases from allergy, cancer, Alzheimer’s disease, and many others. Thus, it is now one of the hottest topics in medical science.

The primary focus of inflammation is to remove the source of disturbance and restore the tissue homeostasis [1]. Traditionally, innate immunity is the rapid response in the form of phagocytosis, whereas the adaptive immunity is antigen-dependent and characterized by an immunological memory which enables mounting a more efficient immune response on subsequent exposure to the same antigen. The hallmark of this inflammatory response is the production of soluble signaling molecules called cytokines. Inflammation involves a cascade of complex events. The first step is the detection of an infection signal [2] and/or damaged tissues [3] which is mediated by the pathogen-associated molecular patterns (PAMPs) that specifically recognize the molecules expressed by the pathogens. Damage-associated molecular patterns (DAMPs), are endogenous molecules that recognize cells committed to death and activate the immune system via the interaction with pattern recognition receptors (PRRs). In response to the successful recognition of these signals, either transmembrane Toll-like receptors (TLRs) or the inflammasomes activate specific immune signaling pathways, resulting in the subsequent activation of nuclear factor kappa-light-chain-enhancer of activated B (NF-κB). As a consequence, NF-κB dissociates from IκB and translocates to the nucleus, where transcription process is up-regulated. These events further lead to the next stage of the cascade, which involves the secretion of pro-inflammatory cytokines, such as interleukin-1-beta (IL-1β), IL-6, tumor necrosis factor-alpha (TNF-α) and others [4]. These then recruit immune cells, such as monocytes and neutrophils at the site of injury resulting in the generation of reactive oxygen and nitrogen species (ROS, RNS) that damage macromolecules including proteins and DNA. In normal conditions, restoration blocks any further neutrophil recruitment for wound healing and reestablishes tissue homeostasis. In chronic “inflammation” cellular damage risk is multi-fold with sustained inflammatory response that causes tissue injury.

Inflammatory cells such as mast cells, macrophages, monocytes, and lymphocytes release inflammatory mediators including cytokines, chemokines, and prostaglandins which further recruit inflammatory cells to the site of injury resulting in a respiratory burst and elevated oxidative stress. These further recruit macrophages and directly activate NF-κB, Mitogen-Activated Protein Kinase (MAPK), and Janus kinase (JAK)- signal transducer and activator of transcription protein (JAK-STAT) signaling pathways associated with the inflammatory response [5,6]. The activation of transcription factors, such as NF-κB and Nrf2, are key components of inflammation signaling cascades and oxidative stress responses [5]. These transcription factors and their functional interrelation will be described in the following sections.

## 2. Nrf2-Keap1 Signaling Pathway

**Figure 1:**
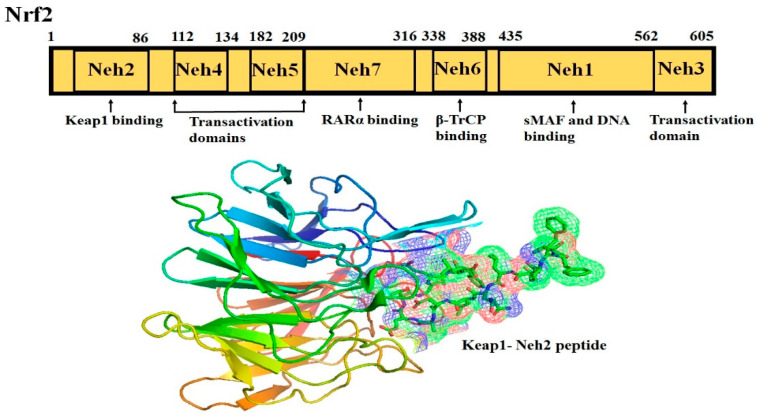
A surface presentation of the Kelch domain (carton) with peptide from Neh2 domain of Nrf2 (sticks in mesh) from crystal structures: 2FLU.

Nrf2 (NF-E2-related factor 2), a member of the Cap’n’collar (CNC) transcription factor family, consists of 605 amino acids and is divided into seven highly conserved functional domains, known as Neh1-Neh7 (Figure 1). The N-terminal domain influences the stability and ubiquitination of Nrf2 by its negative regulator Keap1, while the Neh5 domain is responsible for the cytoplasmic localization of Nrf2 [7]. The Neh1 domain has a cap ‘n’ collar basic-region leucine zipper (bZIP) domain, which regulates DNA-binding [8] and a nuclear localization signal (NLS) that is responsible for the nuclear translocation of Nrf2 [9]. The Neh3, Neh4, and Neh5 are transactivation domains mediating the interaction of Nrf2 with other coactivators [10,11]. Neh6 domain with serine-rich residues is a negative regulatory domain which binds to a β-transducin repeat-containing protein (β-TrCP) leading to Nrf2 ubiquitination [12]. The Neh7 domain inhibits Nrf2-ARE signaling pathway by promoting the binding of Nrf2 to the retinoic X receptor α (RXRα) [13].

The Neh2 domain, an N-terminal regulatory domain, contains seven lysine residues that influence the ubiquitin conjugation [14] and two peptide binding motifs (ETGE and DLG) that regulate Nrf2 stability by promoting its binding to different proteins [15]. The ETGE and DLG motifs interact with Keap1, which is a substrate adaptor protein for the Cullin 3- dependent E3 ubiquitin ligase complex that facilitates Nrf2 ubiquitination and its proteasomal degradation under normal physiological conditions [15,16,17].

**Figure 2:**
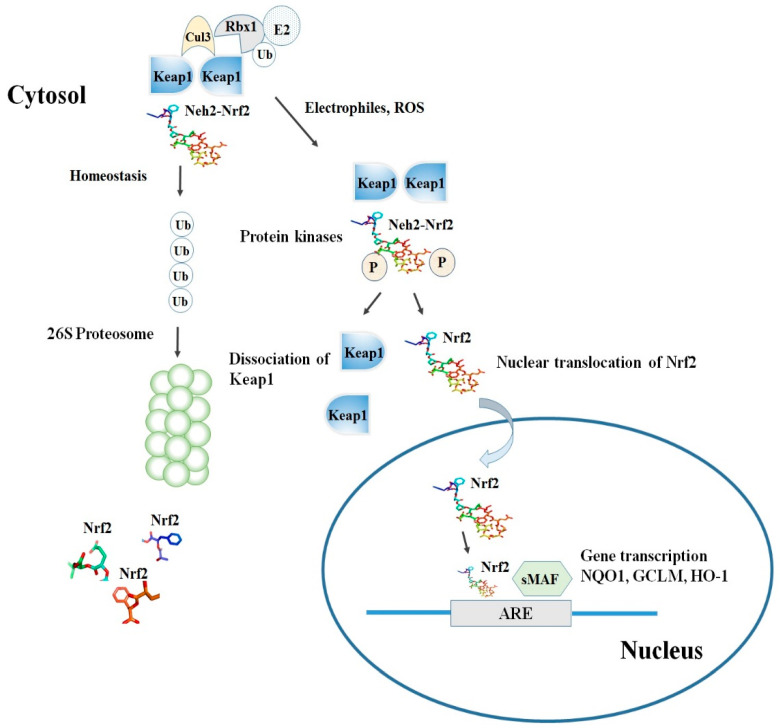
Under normal homeostatic conditions, Keap1 homodimerizes through the N-terminal BTB domain and binds to the cullin-based (Cul3) E3 ligase, forming Keap1-Cul3-RBX1 (Ring box protein-1) E3 ligase complex, leading to Nrf2 ubiquitination and degradation. Under stress (electrophiles or ROS or endoplasmic reticulum (ER) stress) conditions, Nrf2 is released from Keap1-Cul3-RBX1 complex and translocates into the nucleus wherein it heterodimerizes with small Maf proteins (sMaf) and binds to the antioxidant response elements (AREs), leading to the transcription of ARE-driven genes.

Keap1, consisting of 624 amino acids, is a cysteine-rich protein, containing 27 cysteine residues in humans [18]. Keap1 is divided into five domains, an N-terminal region (NTR), a Tramtrack and Bric-à-Brac (BTB) domain, a central intervening region (IVR) with a nuclear export signal (NES) mediating the cytoplasmic localization of Keap1 [19], six Kelch repeats, and a C-terminal domain (CTR) (Li et al., 2004). The BTB domain is responsible for Keap1 homodimerization and its binding to the cullin-based (Cul3) E3 ligase, leading to the formation of Keap1-Cul3-RBX1 (Ring box protein-1) E3 ligase complex [20], whereas Kelch repeats are thought to mediate the binding of Keap1 to Nrf2 and p62 (Figure 2) [21,22].

Among the others, the cysteine residues C151, C273, and C288 are highly reactive and susceptible of covalent modifications by ROS, RNS, H2S and other electrophiles. In this respect, the S-sulfenylation, the S-nitrosylation and the S-sulfhydration of these critical residues were shown to induce conformational changes of Keap1 that ultimately promote the dissociation of Nrf2 and its stabilization [23,24,25]. Although the exact mechanism of the Nrf2-Keap1 interaction is still unknown, two models were reported in the literature to explain the regulation of Nrf2 stability. The first, also known as the ‘‘hinge and latch’’ model, postulates that Keap1 interaction with the ETGE domain acts as a hinge while a weaker interaction with the DLG motif acts as a latch [26].

When specific thiol residues are modified by electrophiles, the DLG motif dissociates from Keap1 causing a disruption in the alignment of Nrf2 lysine residues that ultimately prevent its ubiquitination (Figure 2) [23,27]. Consequently, Nrf2 is released from Keap1-Cul3-RBX1 complex and translocates into the nucleus wherein it heterodimerizes with small Maf proteins (sMaf). After release, Nrf2 binds to EpRE in the presence of small Maf, and up-regulates electrophile response element (EpRE)-mediated transcription [28]. This further activates the transcription of a battery of genes containing an antioxidant response element (ARE) within their promoter region [27]. In addition, the carboxy-terminal domain of Neh3 can interact with the transcription coactivator, CHD6 (chromo-ATPase/helicase DNA-binding protein) [10] while Neh4 and Neh5 can interact with another transcriptional co-activator, CBP (cAMP-response- element-binding protein-binding protein) [11]. Finally, Neh4 and Neh5 can also bind to the nuclear cofactor RAC3/AIB1/SRC-3, further expanding the list of Nrf2 functional interactors, to promote the expression of Nrf2-targeted ARE genes [29]. Interestingly, as shown by several studies, thiol modifications of specifically C151 in the BTB domain might represent a stress sensing-mechanism that prevents the Keap1-Cul3 interactions and thus facilitates Nrf2 activation in response to adverse conditions [30].

The second model, also known as Keap1-independent regulation, postulates that in normal conditions, Neh6 domain binds to the DSGIS and DSAPGS motifs of the β-TrCP (Beta-transducin repeats-containing protein), which in turn is a substrate receptor for the Skp1-Cul1-Rbx1/Roc1 ubiquitin ligase complex that drivesNrf2 ubiquitination [31]. The phosphorylation of Nrf2 in the Neh6 domain by glycogen synthase kinase-3 regulates the recognition of Neh6 domain by β-TrCP [32].

## 3. Nrf2 and NF-ĸB Interplay in the Regulation of Cellular Redox Pathway

It is assumed that Nrf2 and NF-κB signaling pathways cooperate to maintain the physiological homeostasis of cellular redox status and to regulate the cellular response to stress and inflammation. However, the molecular mechanisms underlying this functional interaction appear to be cell type and tissue specific, and are still under elucidation [33]. However, the observation that Nrf2-knockout mice exhibit a strong down-regulation of phase II enzymes expression, suggests that the regulation of these genes is strictly dependent on Nrf2 [34]. In addition, other studies also indicate that Nrf2 system is essential for the expression of phase I and phase III xenobiotic transporters [35,36].

NF-ĸB is a complex protein system constituted by transcription factors that regulate the expression of genes influencing innate and adaptive immunity, inflammation, oxidative stress responses, and B-cell development. NF-kB activation involves both the canonical and non-canonical pathways. In the canonical pathway, NF-ĸB is sequestered as inactive form in the cytoplasm by its inhibitory proteins, including IĸB family members and other ankirin repeats-containing regulators. In presence of specific stimuli such as proinflammatory cytokines, PAMPs, oxidative stress and growth factors, the IĸB kinase complex gets activated and phosphorylates IĸB proteins, ultimately leading to their ubiquitination and proteasomal degradation. As a consequence, p50/RelA and p50/c-RelNF-κB dimers are free to translocate into the nucleus and transactivate multiple target genes. All NF-κB proteins contain a conserved Rel homology domain responsible for dimerization and DNA-binding [37]. NF-κB proteins can be classified into two groups based on the presence or absence of a transactivation domain. RelA (p65), RelB, and c-Rel contain transactivation domains, whereas p50 and p52 do not, and thus, they require heterodimerization with the Rel proteins to induce transcription [37].

**Figure 3:**
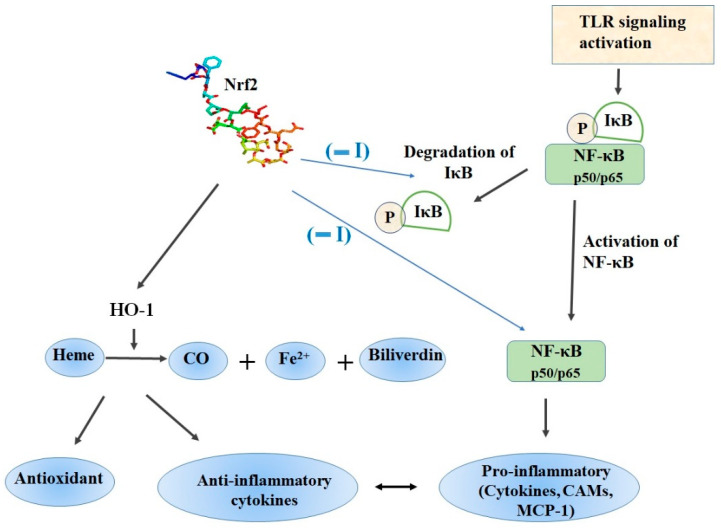
Interplay of Nrf2/HO-1 axis and NF-κB in inflammation. Upon TLR signaling, NF-κB is liberated from IκB, by phosphorylation by the IKK complex. NF-κB then translocates into the nucleus and induces the expression of proinflammatory cytokines, CAMs and other molecules. Nrf2 induces increase in the cellular HO-1 expression and inhibits oxidative stress-mediated NF-κB activation and blocks the degradation of IκB-α. In addition, Nrf2 negatively regulates the IκB-α proteasomal degradation and nuclear translocation of NF-κB.

Nrf2/ARE signaling plays a crucial role in the protection against oxidative stress and is responsible for the maintenance of homeostasis and redox balance in cells and tissues [15]. In contrast, NF-κB is also a redox-regulated transcription factor, which regulates inflammatory responses and cellular injury [38]. After nuclear translocation, NF-κB induces the expression of proinflammatory cytokines (IL-1, IL-6, TNF-α), COX-2, iNOS, vascular adhesion molecules, and others (Figure 3) [38]. From a functional perspective, Nrf2 negatively controls the NF-κB signaling pathway by multiple mechanisms. Firstly, Nrf2 inhibits oxidative stress-mediated NF-κB activation by decreasing the intracellular ROS levels [39]. In addition, Nrf2 prevents the IκB-α proteasomal degradation and inhibits nuclear translocation of NF-κB [40]. Up-regulation of Nrf2 induces increase in the cellular HO-1 levels and subsequent increase in phase II enzymes expression blocks the degradation of IκB-α [41]. On the other hand, NF-κB decreases free CBP, which is a transcriptional co-activator of Nrf2 by competing with CH1-KIX domain of CBP while also promotes phosphorylation of p65 at Ser276 which in turn prevents CBP from binding to Nrf2 [42].

Accumulating evidence also suggests that Nrf2 counteracts the NF-κB-driven inflammatory response by competing with transcription co-activator cAMP response element (CREB) binding protein (CBP) [43,44,45]. The CBP-p300 complex is responsible for the acetylation of histones and exposes DNA for transcriptional machinery assembly. Apart from this, the CBP-p300 complex also acetylates lysine residues of non-histone proteins, including Nrf2 and p65 [8,42]. CBP was also reported to interact with Neh4 and Neh5 domains of Nrf2, leading to the acetylation of the Neh1 domain which is responsible for DNA-binding [42]. Since, CBP also preferentially interacts with phosphorylated p65 at Ser^276^, the overexpression of p65 limits the availability of CBP for Nrf2 interaction [42]. Accordingly, knockdown of p65 promotes Nrf2 complex formation with CBP [42]. Previous studies revealed that the transcription factor, NF-κB(p65) antagonizes the Nrf2-ARE signaling pathway by recruiting MafK-associated HDAC3 activity to the AREs-enriched HO-1 E1 enhancer and thereby deacetylates CBP suppressing its co-activator activity [46,47]. This event is also responsible for local “histone hypoacetylation” leading to repressed transcription and consequently, decreased induction of ARE-containing genes [48]. Thus, as a whole, Nrf2 transcription is down-regulated due to CBP sequestration as well as the abrogation of CBP co-activator activity caused by its deacetylation [46]. It was reported that the physical association of the N-terminal region of the p65 subunit of NF-κB with Keap1 can inhibit the Nrf-2 pathway [45]. However, numerous studies showed that different pathological sources of exogenous or endogenous stress such as lipopolysaccharide, oxidized LDL, pro-atherogenic oscillatory stress, smoke, shear stress, and other stress signals often activate both NF-κB and Nrf2-ARE signaling [49,50,51,52,53,54,55,56].

A recent paper studying inflammation in prostate cancer and castration-resistant prostate cancer showed that antioxidant and anti-inflammatory agents such as sulforaphane and curcumin can activate Nrf-2 and induces the nuclear accumulation of p120-Nrf1 which in turn significantly inhibits NF-κB DNA-binding activity by decreasing the transactivation of androgen receptor signaling [56].

Interestingly, some Nrf2 target genes such as NADPH quinone oxidoreductase I (NQO1), glutamate-cysteine ligase catalytic subunit (GCLC) and glutamate-cysteine ligase modifier subunit (GCLM) are also believed to possess NF-κB binding site. Thus, it is plausible that these two signaling pathways may cooperate in some specific cases, for example promoting chemoresistance of acute myeloid leukemia cells in response to bortezomib treatment [57,58]. This could be the reason compounds targeting NF-κB signaling conversely activate the Nrf2 pathway [59,60]. However, research focused on the potential impact of NQO1 on the NF-κB signaling led to conflicting results. One study suggested that the overexpression of NQO1 suppresses the expression of tumor necrosis factor (TNF)-α and IL-1 in LPS-stimulated THP-1 cells independently from NF-κB [61]. In contrast however, another study indicated that in NQO1 deficient mice NF-κB DNA-binding was inhibited in bone marrow, spleen, and thymus on LPS stimulation [62]. Similarly, nuclear thioredoxin (Trx) can modulate NF-κB activity via the reduction of cysteine residues on p50 and thus prevent DNA-binding [63,64,65,66] while cytoplasmic Trx prevents IκB degradation [67]. Cytoplasmic binding of Trx1 to IκB blocks its degradation and subsequently inhibits nuclear translocation of NF-κB, thereby decreasing its transcriptional activity [67]. However, the mechanism by which IκB is regulated by Trx1 was not yet elucidated.

Emerging evidence also revealed that NF-κB induction can promote Nrf2 activity via the small GTPase Ras-related C3 botulinum toxin substrate 1 (Rac1) protein (Cuadrado et al., 2014). For example, it was shown that the constitutively active form of Rac1 was able to induce the activation and the nuclear translocation of Nrf2^ΔETGE^-V5, which lacks four residues (ETGE) required for its recognition by Keap1, suggesting that Nrf2 induction through Rac1 actually occurs in a Keap1-independent manner (Cuadrado et al., 2014). In turn, Nrf2 suppresses Rac1-dependent activation of NF-ĸB pathway, whereas Nrf2 deficient cells promote NF-ĸB dependent inflammatory markers, suggesting the existence of a negative feedback mechanism that might regulate the intensity of Nrf2 activation under specific circumstances [68]. Indeed, LPS was shown to activate Rac1 which in turn up-regulates HO-1 expression through the NF-κB-dependent induction of Nrf2 signaling. Furthermore, the antioxidant enzyme HO-1, in turn, was found to suppress the NF-κB dependent inflammatory activity, indicating the crucial role of Nrf2 in the reduction of oxidative stress [68].

Some other reports stated that Nrf2 activation attenuated oxidative stress and neuronal inflammation through the suppression of the NOX4/ROS/NF-ĸB pathway [69,70]. Interestingly, Nrf2 overexpression modulates only targets lying downstream NF-κB in human aortic endothelial cells, since the expression of MCP-1, VCAM-1 and TNF-α was abrogated, without affecting NF-κB activity or IκB degradation [71,72,73,74].

Furthermore, sulforaphane, a potent Nrf2 inducer, was shown to prevent DNA-binding of NF-κB and to downmodulate the expression of NO, PGE_2_, and TNF-α in macrophages, while it failed to prevent LPS-induced IkB degradation or NF-kB nuclear translocation. Despite the role of NRF2 was not investigated, the authors proposed that sulforaphane might induce thiol modifications in the NF-kB subunits to impair its DNA-binding capacity [75]. In support of this evidence, several other reports showed enhanced NF-κB activation in Nrf2 knockout mice subdued to different stimuli such as LPS [76], ovalbumin [77], traumatic brain injury [78] and respiratory syncytial virus [79].

Nrf2 depletion in human monocytes reportedly enhanced TNF-induced inflammatory cytokines [80]. Mechanistically, TNF-induced sustained activation of Nrf2 and target genes expression through a TNFR1-dependent mechanism causing autocrine TNF production. The same study reported that Nrf2 silencing enhanced both TNF-α -induced proinflammatory genes expression as well as p50 and p65 DNA-binding, indicating a negative regulation of Nrf2 on NF-kB activation [80]. In agreement with these findings, disruption of Nrf2 signaling in knockout mice was shown to cause enhanced sensitivity to septic shock and lung inflammation in response to LPS or TNF-α. Accordingly, further analysis revealed that Nrf2-deficient MEFs (mouse embryonic fibroblasts) exhibited increased NF-kB activation in response to LPS and TNF-α treatment, suggesting that Nrf2 could suppress the inflammation induced by these stimuli blocking the induction of NF-kB [76].

In marked contrast; however, Nrf2 knockout in murine fibroblasts was shown to suppress p50 and p65 levels and enhance c-Rel protein content, whereas livers of Nrf2 knockout mice exhibited greatly reduced p65 levels. Based on this evidence the authors proposed that Nrf2 was actually required for the expression of NF-κB family members [81]. Taken together, these data point out the existence of a functional cross-talk between NF-kB and Nrf2 signaling while also highlight that this complex interplay can enhance or impair the inflammatory response, depending on the specific context and stimuli.

Oxidative stress has long been implicated in many pathological conditions and ROS accumulation plays a crucial role in the progression of inflammatory responses. Since, the mitochondrion is a primary site for ROS production under physiological and pathological conditions, a series of studies demonstrated the crucial role of the Nrf2 signaling pathway in cytosolic and mitochondrial ROS production [82]. For instance, NOX-2 (NADPH oxidase 2) activity is increased in Nrf2 deficient embryonic fibroblast cells and unrestricted Nrf2 activation in *Keap1* knockout fibroblasts leads to increased NOX-4 activity [82]. In addition, up-regulated transcription of all the genes (GSTα2, NQO1, TrxR1, and GCLC) on ectopic Nox4 expression was observed to be ablated in cardiomyocytes of Nrf2-null mice, suggesting the essential role of Nrf2-Keap1 signaling in glutathione redox signaling [83]. A recent report shows that Nrf2 is associated with the outer mitochondrial membrane and protects mitochondria from oxidative stress, whereas this protective effect was absent in Nrf2 knockout mice [84]. Interestingly, Nrf2 was also recently found to protect mitochondria in response to oxidative stress suggesting a potential link between Nrf2 with mitochondrial outer membrane, despite, the exact molecular mechanisms through which Nrf2 interacts with the mitochondrial outer membrane and thus confers protection against oxidative insults, remains to be elucidated [84].

Nrf2-regulated target genes, NQO1 and NQO2 are two cytosolic flavoproteins catalyzing the two-electron mediated reduction of quinones to hydroquinones [85]. This catalytic reaction competes with the one-electron reduction reaction catalyzed by cytochrome P450 reductases, which generates highly reactive semiquinones [86]. Furthermore, NQO1 also maintains the reduced form of CoQ_9_ and CoQ_10_ inside the unilamellar or multilamellar vesicles, thereby protecting the plasma membrane from lipid peroxidation and free radicals. Knockout of NQO1 is associated with high susceptibility to immune disorders [87,88]. Moreover, the double knockout of both NQO1 and NQO2 strongly enhances the infiltration of neutrophils and macrophages in bronchial-associated lymphoid tissue, leading to an increase in pro-inflammatory cytokines in lung macrophages [89]. Another report showed that the induction of NQO1 by several synthetic triterpenoids resulted in the decreased expression of the pro-inflammatory inducible nitric oxide synthase (iNOS) in Hepa1c1c7 murine hepatoma cells, an effect that was strictly Nrf2-dependent and related to the direct interaction with Keap1 thiol groups [90].

Nrf2 directly modulates the ROS and RNS by regulating the levels of superoxide and peroxides through the induction of antioxidant enzymes such as superoxide dismutase, glutathione peroxidase and peroxiredoxin members [91,92,93]. In response to oxidative stress, Nrf2 translocates to the nucleus and activates the transcription of several antioxidant genes, such as glutathione biosynthetic enzymes (GCLM and GCLC), GSH-dependent antioxidant enzymes (glutathione peroxidase 2, GPX2), and glutathione *S*-transferases (GST) [94]. The Nrf2-dependent regulation of redox homeostasis operates at multiple levels of redox control, one of them involving the regeneration of reduced cofactors and protein-thiol groups from their oxidized counterparts. Well known examples include the conversion of glutathione disulphide (GSSG) into glutathione (GSH) by the glutathione reductase (GSR) [95], the reduction of oxidized thioredoxins by thioredoxin reductase (TrxR) [96] and Prx-SO2H by sulfiredoxin (Srx) [97]. Another mechanism, implies the transcriptional induction of enzymes involved in the generation of reducing factors, as occurs in the biosynthesis of GSH by glutamate-cysteine ligase (catalytic and regulatory) or NADPH by glucose-6-phosphate dehydrogenase (G6PDH) and other metabolic enzymes [98]. Accordingly, Nrf2-deficient mice show impaired expression of glutamate cysteine ligase (GCL) and strong depletion of the GSH levels [76]. As reported by Hartl et al., suppression of GSH, in turn, led to high susceptibility to oxidative stress, up-regulation of pro-inflammatory cytokines and impairment in T-cell responses [99]. Consistently, the overexpression of GCL was found to inhibit TNF-α-induced activation of NF-κB, AP-1, and JNK in rat hepatoma cells [100], while increase in GSH synthesis was shown to down-regulate the NF-κB activation and TNF-α release in LPS-stimulated alveolar type II (AT-II) epithelial cells [101]. Similarly, GSH was also found to down-regulate p38 activation as well as iNOS and COX-2 expression in rat peritoneal macrophages subdued to LPS stimulation [102], indicating that the GSH content can strongly influence the activity and function of molecular and cellular mediators of the inflammatory processes. This is in agreement with previous findings demonstrating that GSH-mediated redox status was indeed able to affect the Th1/Th2 balance while GSH depletion was found to inhibit Th1-associated cytokines release and favor Th2-associated responses [103,104].

In addition to this, several other mechanisms of Nrf2-mediated redox regulation were described. For example, the antioxidant protein thioredoxin (Trx) can be transactivated by hemin (ferriprotoporphyrin IX) through the binding of the Nrf2/small Maf complex to ARE [105]. The thioredoxin (Trx) system is a major intracellular thiol-reducing and ROS-scavenging pathway consisting of three components. Among them, the thioredoxin-interacting protein (TXNIP) acts as a negative regulator that binds to the reduced form of Trx and prevents it from reducing peroxiredoxin (Prx), thereby inhibiting Trx mediating function. In this context, Nrf2 was shown to inactivate the TXNIP expression and to suppress its induction under high glucose conditions through the interaction with ARE of the TXNIP promoter, which in turn inhibited the binding of MondoA to the carbohydrate response element in presence or absence of glucose [106]. This suggests the critical role of Nrf2 in redox regulation mediated by the TXNIP-Trx axis [106].

Another line of evidence revealed the key role of Nrf2 in cystine/glutamate exchange transport which ultimately regulates the intracellular GSH levels by influencing the uptake of cystine, the oxidized form of cysteine, a limiting substrate of GSH synthesis [107]. The cystine/glutamate exchange transport is found to be mediated by x_c_−system, which consists of two protein components, xCT and a heavy chain of 4F2 antigen [107]. Nrf2 was demonstrated to activate transcription of xCT in response to various stimuli, such as diethyl maleate (DEM), cadmium chloride, arsenite, and lipopolysaccharide, via the electrophile response element (EpRE) [108]. Electrophilic agents including DEM, are thought to promote the dissociation of Nrf2-Keap 1 complex and release of Nrf2 [108].

Another mechanism through which Nrf2 controls the redox balance is by influencing the expression of genes coding for proteins involved in iron storage, transport and metabolism. Iron and oxygen are interconnected and iron essentially acts as a cofactor in oxygen transport, oxidative phosphorylation, and metabolite oxidation reactions. However, excess iron leads to the development of oxygen-derived free radicals. Here, Nrf2 regulates iron metabolism pathway in response to oxidative and electrophilic stress by activating transcription of genes coding for ferritin heavy chain (*Fth1)* and ferritin light chain (*Ftl)* [109], thus increasing ferritin levels [110]. As a result, Nrf2 indirectly promotes iron storage and reduces the intracellular levels of redox-active free iron atoms. Moreover, Nrf2 also regulates labile iron (redox active, exchangeable and chelatable) by altering its transport across the cell membrane by up-regulating the expression of ferroportin (Fpn1) [111], which is a protein responsible for iron export from the cytosol to the extracellular milieu [112]. Similarly, Nrf2 plays a crucial role in the gene expression of metallothionein, a cysteine-rich metal-binding protein and another downstream target implied in the antioxidant pathway [113].

In response to the oxidative stress, cells induce another set of antioxidant enzyme including heme oxygenase-1 (HMOX1) and thioredoxin reductase-1 (TrxR1). Here, Nrf2 confers an additional layer of regulation for the protection against oxidative stress [114]. In the same study, oxidative stress triggered by hemin treatment-induced both proteasomal degradation and displacement of BACH1, a transcriptional repressor, from *HMOX1* gene enhancers, which in turn allowed nuclear Nrf2 binding to the ARE motifs and subsequent transactivation of the *HMOX1 gene* [114]. In contrast, TrxR1 appeared to be directly regulated by Nrf2 without requiring previous BACH1 inactivation [114].

Since autophagy is reciprocally linked to oxidative stress, several studies also support the notion that Nrf2 can play a role in the regulation of autophagy. A crucial link between autophagy and Nrf2 is represented by p62, a ubiquitin-binding protein acting as an autophagy cargo receptor [115]. Under normal physiological conditions, p62 is constantly degraded during autophagy; however, under pathological oxidative stress conditions p62 accumulates and competitively binds to Keap1, thus causing the release of Nrf2 and the activation of its downstream target genes [116].

These findings hint that in response to oxidative stress, up-regulation of Nrf2 signaling activates a complex antioxidant response and maintains the redox homeostasis through the regulation of multiple mechanisms.

## 4. Cross-Talk between Nrf2 and Inflammation

Nrf2 is part of a multilayered network that plays a key role in redox homeostasis as well as in inflammation. Next, we will discuss the molecular mechanisms through which Nrf2 is regarded to influence both innate and adaptive immunity.

### 4.1. Role of Nrf2/HO-1 Axis in Inflammation

Heme oxygenase-1 (HO-1, HMOX1, EC 1.14.99.3), is an inducible 32 kDa protein and catalyzes the rate-limiting step of oxidative heme degradation. During this process, heme is converted into three bioactive products namely free iron, carbon monoxide (CO) and biliverdin, which is rapidly converted to bilirubin and plays crucial roles in inflammation, apoptosis and oxidative stress (Figure 3) [117]. Consistently with other antioxidant proteins, Nrf2 directly controls the expression of the *HMOX1* gene codying for the HO-1 enzyme. The critical role of the Nrf2 mediated HO-1 expression for the anti-inflammatory activity was substantiated in a series of in vitro and in vivo experiments.

In a study focused on macrophage polarization, gene expression profiling showed that in vitro M-CSF-polarized macrophages were characterized by preferential expression of both CD163 and HO-1. Further analysis revealed that the experimental induction of HO-1 activity by cobalt protoporphyrin treatment resulted in the increased release of IL-10 in M2 macrophages, suggesting that the CD136/HO-1/IL-10 axis can influence the anti-inflammatory and regulatory functions of M2-macrophages [118]. In addition, the levels of CD206^+^ and HO-1 in M2 macrophages were found to correlate with the anti-inflammatory activity in diabetes-associated gastric pathology [119]. Similarly, hemin-mediated induction of HO-1 in macrophages contributed to anti-inflammatory activity in acute pancreatitis [120]. These findings implicated the therapeutic potential of targeted modulation of the heme-HO-1 system in macrophages.

After silencing *Nrf2* via knock-out, the expression of COX-2, iNOS, IL-6, and TNF-α were dramatically increased in Parkinson’s disease [121]. Similarly, the Nrf2 activation led to a decrease in expressions of COX-2 and iNOS in vascular smooth muscle cells [122]. The enhanced expression of inflammatory proteins (COX-2 and iNOS,) and inflammatory cytokines (TNF-α and IL-6) was found to be accompanied by a concurrent increase in HO-1 and NQO1 content, implicating the role of Nrf2/HO-1 axis in inflammation. HO-1 can be rapidly induced by various types of oxidative/electrophilic stress which in turn can inhibit ROS and NO production in immune cells [123].

*HO-1* knockout studies revealed a novel mechanism of the Nrf2/HO-1 axis in inflammation. For instance, HO-1 deficiency in human and mice enhanced systemic inflammation, impaired the fibrinolysis system and was accompanied by anemia, intravascular hemolysis and vascular endothelial injury [124,125,126]. Activation of Nrf2/HO-1 axis was found to reduce lipid peroxidation and TNF-*α* and IL-6 levels, and consequently to attenuate the inflammatory reaction in a tight junction dysfunction model [127]. Also, Taurine chloramine (TauCl), a product of activated neutrophils, was shown to induce the clearance of apoptotic cells by phagocytes, also known as efferocytosis, when injected into macrophages. Importantly, this process was abolished in Nrf2*/HO-1* knockout macrophages, and in those cases, inflammation was resolved by Nrf2-mediated HO-1 up-regulation and subsequent production of CO [128]. Moreover, it was reported that Nrf2 mediated HO-1 up-regulation along with PPAR*γ* ligands are responsible for the resolution of inflammation in chronic obstructive pulmonary disease [129].

Furthermore, the metabolites of heme degradation including CO and bilirubin also accelerate the resolution of oxidative stress and inflammation [117,130]. CO prevented bone destruction in collagen-induced arthritis and reduced cytokine levels in hind-limb ischemia-reperfusion injury [131,132]. Similar results were also obtained with bilirubin which showed amelioration of autoimmune encephalomyelitis [133], autoimmune hepatitis [134], endothelial activation and dysfunction [135]. Another study reported that bilirubin could exert a protective effect against endotoxic shock by preventing the expression of iNOS and thus the generation of NO [136]. Collectively, these findings suggest that Nrf2 largely mediates the anti-inflammatory action of HO-1.

Accumulating evidence indicates that the role of Nrf2/HO-1 signaling may be stimulus and cell type-specific [137,138]. It is already known that phosphorylation of Nrf2, Nrf2-Keap1 dissociation, and Nrf2/HO-1 signaling pathway is functionally interrelated (Figure 3). Indeed, a quite recent report showed that the Nrf2/HO-1 axis mediated the protective effect of isorhamnetin against H2O2 treatment in C2C12 myoblasts and that ERK (extracellular-signal-regulated-kinase) activation was required for the phosphorylation of Nrf2 and Nrf2/HO-1 signaling, whereas no involvement of PI3K, JNK, and p38 MAPK was observed [139]. In contrast, another report revealed that the MAPK-dependent phosphorylation of Nrf2 is required for its nuclear translocation in HepG2 hepatoma cells subdued to treatment with PDTC [140]. The authors demonstrated that the initial phosphorylation of Nrf2 on MAPK sites releases Nrf2 from Keap1 and this was followed by nuclear translocation and finally, DNA-binding [140]. Furthermore, nuclear translocation and DNA-binding also requires phosphorylation. Therefore, Erk-mediated phosphorylation of Nrf2 could be a prerequisite for its translocation despite the exact mechanism by underlying this process is yet to be elucidated.

### 4.2. Functional Interaction of Nrf2 with Inflammatory Mediators

It is well established that inflammation is a complex interplay of different inflammatory cells that release several signaling molecules such as arachidonic acid derivatives (leukotrienes and prostaglandins), phospholipid mediators (platelet-activating factor), and cytokines (interleukins and other bioresponse modifiers) which are responsible for the inflammatory response. Cytokines are low molecular weight extracellular polypeptides and glycoproteins that regulate homeostasis and signal-dependent functioning of immune cells. Cytokines are key players of inflammation and include interleukins (ILs), chemokines, interferons (IFNs), tumor necrosis factor (TNF), colony-stimulating factor (CSF), and growth factors (GFs). A wide array of inflammatory signaling responses is involved and cytokines can be broadly classified as pro-inflammatory or anti-inflammatory mediators.

#### 4.2.1. Role of Nrf2 in the Regulation of Cytokines

LPS stimulation induces exaggerated massive expression of inflammatory mediators including cytokines, chemokines, and adhesion molecules in Nrf2 knockout mice [76]. For many years, it was believed that macrophages polarize into a pro-inflammatory ‘M1′ phenotype, promoting the secretion of pro-inflammatory mediators such as TNF, IL-1 and IL-6 and small molecule mediators of inflammation such as ROS, RNS and prostanoids but conversely inducing a simultaneous decrease in anti-inflammatory cytokines such as transforming growth factor (TGF)-α, IL-4, IL-13, and IL-10 [141]. The activation of Nrf2 signaling represents an interesting alternative method to direct resolution of inflammation. It was reported that activation of Nrf2 has the potential to inhibit LPS-induced up-regulation of pro-inflammatory cytokines including *IL-6* and *IL-1β*, through the ROS-independent inhibition [142]. This connection was further corroborated by the observation that Nrf2 negatively regulates the NF-κB-mediated transcription of pro-inflammatory cytokine genes [142]. The binding sites of Nrf2 near the *IL-6* and *IL-1β* promoters coincides with the common binding regions of pro-inflammatory transcription factors, such as p65, C/EBPβ and c-Jun, suggesting that the recruitment of Nrf2 in this region might involve the interaction with other factors. This type of association was firstly suggested by studies reporting that p65 is responsible for the regulation of Nrf2 signaling [142]. Other co-activators include CBP, and Mediator complex MED16 and MED24, which were also reportedly found to be associated with Nrf2 in LPS-stimulated cells [143,144]. Also, histone-modifying enzymes, such as CBP/p300, were identified as specific cofactors recruited to the promoter region upon DNA-binding of a signal-specific transcription factor, an event followed by the recruitment of Mediator complex and the subsequent expression of Nrf2 target genes [145]. It is plausible that the recruitment of Nrf2 in regulatory regions of pro-inflammatory cytokine genes might abrogate their transcription by interfering with their transcription factors. This hypothesis is supported by evidence showing that the Nrf2 inducer Tecfidera, inhibited the IL-6 and IL-1 gene expression in experimental models of multiple sclerosis and other autoimmune diseases [146,147,148,149]. A significant reduction in Th1 and Th17 cytokines including IL-6 was observed upon Nrf2 activation induced by derivatives of the triterpene oleanolic acid, namely CDDO-trifluoroethyl-amide (CDDO-TFEA) mediating the suppression of *IL-6* gene expression [150].

A study in *Nrf2* knock-down peritoneal neutrophils also indicated that LPS stimulation was followed by a significant elevation in cytokines (TNF-α, IL-6) and chemokines such as monocyte chemotactic protein-1 (MCP-1) and macrophage inflammatory protein 2 (Mip2) as well as increased ROS levels [151]. In addition, it was also observed that the expression of GCLC, GCLM, and NQO1 was significantly up-regulated in wild-type neutrophils as compared with Nrf2 deficient cells [151]. However, pretreatment of peritoneal neutrophils with the Nrf2 activator CDDO, induced Nrf2-dependent antioxidant genes and attenuated changes in proinflammatory cytokines and chemokines in the lungs [151]. Taken together, these results suggest that oxidative regulation by Nrf2 is responsible for LPS-TLR4 signaling and thereby is crucial for the innate immune response in neutrophils and macrophages. Similarly, Nrf2-dependent HO-1 expression was found to negatively regulate the NF-ĸB and MCP-1 secretion levels in human umbilical vein endothelial cells (HUVECs) upon TNF-α treatment [152].

Some interesting studies conducted on myeloid-derived suppressor cells (MDSCs) demonstrated that the Nrf2 antitumor activity involves the suppression of ROS levels and tumor metastasis caused by impaired IL-6 secretion in MDSCs [153,154]. The ROS and RNS generation was reported to suppress the activation and the proliferation of CD8^+^ T-cells by inducing modifications in the T-cell receptor-CD8 complex upon the surface of CD8^+^ T-cells due to the production of peroxynitrite in MDSCs [155]. These changes disrupted the interaction between major histocompatibility complex class I molecules and the antigen-presenting cells [155], suggesting that the immunological response was implicated in the anti-metastatic activity of Nrf2.

It was also reported that Nrf2 activation by *Keap1* knockout protects from obesity-induced diabetes by improving both insulin secretion and insulin resistance, thus resulting in the prevention of hyperglycemia [156]. Another report demonstrated that the improvement of insulin secretion and protection in type-2 diabetes was associated with the suppression of IL-1 and IL-1 receptor [157].

#### 4.2.2. Role of Nrf2 in the Regulation of Cell Adhesion Molecules

In the pathological inflammatory process, IL-1 and TNF-α, reportedly up-regulate the expression of cell adhesion molecules (CAMs), such as intercellular adhesion molecule-1 (ICAM-1) and vascular cell adhesion molecule-1 (VCAM-1) on the surface of endothelial cells, macrophages, and lymphocytes. All these molecules are responsible for leukocyte recruitment and cytokine production resulting in the tissue inflammation and oxidative stress [158,159]. Based on current knowledge, it is conceivable that Nrf2 might serve as a regulatory factor affecting the expression of cell adhesion molecules (CAMs). Indeed, Keap1 knockdown in HUVECs was shown to significantly attenuate the TNF-α induced increase in the expression of ICAM-1 and VCAM-1, suggesting the crucial role of Nrf2 in the vascular inflammatory responses [160]. Consistently, in response to LPS- administration in mice, Keap1 siRNA resulted in a significant and Nrf2-dependent down-regulation in the levels of TNF-α, iNOS, and several adhesion molecules [160]. On the other hand, *Keap1* knockdown enhanced the expression of HO-1, GCL, and peroxiredoxin-1 (Prx1) in macrophage [160]. Knockdown of *Nrf2* was also shown to stimulate NFκB activation, TNF-α, IL-1β, and IL-6 secretion and to promote the over-expression of ICAM-1 in the brain following traumatic brain injury [161].

Similarly, studies on vascular inflammation and atherosclerosis models revealed that activated Nrf2 can prevent the pro-inflammatory state of vascular endothelial cells by suppressing the p38–VCAM-1 signaling [162]. Here, Nrf2 inactivated p38 by inhibiting MKK3/6 signaling pathway and by inducing the activity of a negative regulator MKP-1 [162]. Moreover, MKP-1 is preferentially expressed by endothelial cells in aorta region where it inhibits p38–VCAM-1 signaling [163]. In this regard, some studies demonstrated the importance of the antioxidant enzyme HO-1, suggesting that the anti-inflammatory action of Nrf2 might in part depend on the modulation of the redox homeostasis. For instance, the plant-derived antioxidant 3-hydroxyanthranilic acid (HA), was found to induce HO-1 expression and to inhibit both VCAM-1 expression and NF-kB activation by promoting Nrf2 translocation to suppress inflammation in atherosclerosis [152]. Consistently, the genetic over-expression of HO-1 was found to inhibit TNF-α-mediated VCAM-1 expression in HUVECs [39]. In agreement with this hypothesis, the anti-inflammatory activity of several natural antioxidants such as lycopene, arachidin-1 and others, were shown to involve the inhibition of NF-κB transcription and ICAM-1 and VCAM-1 expression via Nrf2 dependent signaling pathway [41,164,165]. The Nrf2-dependent modulation of adhesion molecules plays also an important role in mediating cancer cells invasion and adaptation to persistent inflammatory stress during tumorigenesis. Indeed, it was reported that in the context of the inflammation associated with carcinogenesis, Nrf2 activation was able to abrogate the expression of E-cadherin, whereas in contrast to slightly increase the expression of L1CAM in pancreatic ductal adenocarcinoma cell lines [166]. Moreover, this report also identified a ARE region located in the promoter region of the E-cadherin gene which was involved in the Nrf2 dependent regulation of E-cadherin expression [166].

These findings indicate that in response to inflammatory stimuli, up-regulation of Nrf2 signaling inhibits the activation of NF-ĸB and the secretion of pro-inflammatory cytokines and chemokines, attenuating also the expressions of CAMs.

In addition to cytokines, chemokines, and CAMs, inflammatory mediators also include enzymes, which play a crucial role in tissue remodeling and inflammatory response. Activated leukocytes and mast cells express a variety of proteolytic enzymes, particularly serine proteases that ultimately modulate an inflammatory response [167].

#### 4.2.3. Role of Nrf2 in the Regulation of Protease/Antiprotease Balance

Since the imbalance between protease and antiprotease levels and oxidative stress are also thought to be an important aspect of inflammation, Nrf2 is believed to play a regulatory mechanism in this aspect as well. In this respect, it was reported that the susceptibility of Nrf2 knock-out mice to emphysema was caused by a reduced antiprotease activity (Ishii et al., 2005). Mechanistically, Nrf2 knock-out mice showed a significant elevation in neutrophil elastase activity, suggesting protease/antiprotease imbalance (Ishii et al., 2005). The same group also revealed that secretory leukoprotease inhibitor (SLPI) levels were significantly suppressed in Nrf2 knock-out mice, whereas the SLPI gene was highly inducible in the lungs of wild-type mice after porcine pancreatic elastase induced emphysema [168]. By controlling the levels of this crucial regulator, Nrf2 is therefore expected to modulate both antiprotease and anti-inflammatory events in lung inflammation. For example, an earlier study showed that SLPI was markedly expressed at the site of inflammation in response to several inflammatory stimuli such as LPS [169] and proinflammatory cytokines [170]. Quite recent data suggest that SLPI might be a direct target of Nrf2 since its promoter was found to contain ARE regions which were responsible for the overexpression of SLPI by Nrf2 agonists in airway epithelial cells in an Nrf2-dependent manner [171,172].

Another type of inflammatory mediators implicated also in the malignant progression is represented by the matrix metalloproteinases (MMPs), zinc-dependent proteolytic enzymes responsible for the degradation of various components of extracellular matrix components. The activities of MMPs are regulated at multiple levels, including Nrf2 signaling. A series of experiments in *Nrf2* knockout mice have demonstrated its protective role in UV-induced inflammation and the regulation of MMPs activities. For instance, MMP-9 activity and expression of MIP-2, a key mediator of neutrophil recruitment as well as Pro-MMP-9 expression were significantly elevated in Nrf2 knockout mice after UVB irradiation, suggesting that the photo-protective effect of Nrf2 was mediated by the suppression of MMPs expression [173]. Furthermore, Nrf2 could influence MMP-1 expression through the MAPK/AP-1 signaling cascades after UVA-induced stimulation in human keratinocytes (HaCaT cells) [174]. The study revealed that Nrf2-activating compounds could reverse the UVA-stimulated increase in phosphorylation of ERK, JNK, p38, c-Jun, and c-Fos [174]. Another study suggested that Nrf2 deficiency could induce the activation of MAPKs, such as JNK, ERK, and p38, in addition to c-Fos in osteoclast differentiation elicited by pro-inflammatory stimuli, suggesting that Nrf2 activation might be beneficial to treat inflammatory bone diseases [175].

Nrf2/HO-1 signaling inhibits the expression of MMP-9 and iNOS in LPS-stimulated macrophages [176] and IL-8, ICAM-1, COX-2 and MMP-7 expression in TNF-α-stimulated human intestinal epithelial HT-29 cells as well as in colonic mucosal tissue [177]. In addition, Nrf2 deficient mice subdued to spinal cord injury show significant elevation in NF-*κ*B activation, TNF-*α* production, and expression of MMP-9 as compared with wild-type controls [178]. Similarly, in osteoarthritis model, Nrf2-knockout induces proinflammatory cytokines (TNF-α, IL-6, and IL-1β), MMP1, MMP3, and MMP13 expressions in chondrosarcoma cells [179]. In the treatment of diabetic skin ulcers, knockdown of Nrf2 shows delayed wound healing, which is attributed to oxidative DNA damage, reduced TGF-β1 levels, high MMP9 expression and increased apoptosis [180].

Nrf2 was also shown to modulate the expression of CD36 in macrophages. CD36 is a class B transmembrane scavenger receptor expressed by macrophages, microvascular endothelial cells, platelets, and epithelial cells [181]. CD36 is a major endocytic receptor for oxidized lipoproteins and is responsible for the uptake of oxidized LDLs (oxLDLs) by macrophages [181]. In macrophages, CD36 is reportedly up-regulated by oxLDL [182] and oxLDL induce the activation of Nrf2 [183]. The lack of induction of CD36 in Nrf2 knockout macrophages was found to be associated with a reduced accumulation of cholesterol, suggesting a role for the Nrf2 signaling pathway in modulating oxLDL uptake via CD36 [183]. Some other studies also indicate elevation in the expression of CD36 via Nrf2 activation in atherogenesis [184,185,186]. In an intracerebral hemorrhage, the Nrf2 activator sulforaphane induced CD36 expression in the affected brain and improved hematoma clearance in wild-type but not in Nrf2 knockout mice [187].

The inflammatory response also requires a systematic interaction of inflammatory enzymes known as cyclooxygenases (COX) which catalyze the formation of prostaglandins, thromboxane, and levuloglandins [188]. Among all the COX enzymes, COX-2 is mainly involved in acute inflammation [189]. Initially, COX-2 is mainly expressed by macrophages and is up-regulated in response to growth factors or inflammatory stimuli. A potential role of Nrf2 in repressing COX signaling is shown by the fact that the expression of COX-2, iNOS, and proinflammatory cytokines is significantly enhanced in *Nrf2* knockout mice compared to their wild-type counterpart [190,191]. Similarly, a significant increase in oxidative stress and expression of COX-2, iNOS, IL-6, and TNF-α was observed after 1-methyl-4-phenyl-1,2,3,6-tetrahydropyridine stimulation in *Nrf2*-knockout mice [121]. Likewise, some studies also show that the anti-inflammatory role of Nrf2 was reportedly mediated by COX2/15d-PGJ_2_ signaling pathway in experimental pleurisy and acute lung injury models [192,193].

Cyclopentenone PGs (*cyPGs*) such as 15-deoxy-Δ^12,14^-prostaglandin J_2_ (15d-PGJ_2_) were also found to modulate Nrf2 signaling and to induce GST in peritoneal macrophages. Here, it was postulated that the presence of highly reactive α,β-unsaturated carbonyl group might confer to cyPGs the ability to form Michael adducts with nucleophilic molecules and to induce covalent modifications of target proteins, a mechanism that might be responsible for the activation of Nrf2 [192]. 15d-PGJ_2_ was also reported to induce the expression of HO-1 in a Nrf2 dependent manner [194]. Since the redox-sensitive cysteines are highly susceptible to adduct formation with electrophiles, CyPGs including 15d-PGJ_2_ can directly bind to the cysteines within the Keap1 IVR region, resulting in the activation of Nrf2/ARE signaling pathway [195,196,197]. Moreover, the anti-inflammatory activity of 15d-PGJ_2_ against acute lung injury was shown to be mediated by the induction of the Nrf2 pathway and this protective effect was abolished in *Nrf2* knockout mice [192]. Similarly, pretreatment with Nrf2 activators such as sulforaphane, can exert anti-inflammatory effects by suppressing the expression of proinflammatory cytokines, COX-2, and iNOS in peritoneal macrophages, while this effect was abolished in *Nrf2* knockout mice [198]. In human aortic endothelial cells (HAECs), 15d-PGJ_2_ activates Nrf2 and induces Nrf2 target genes expression [196]. COX-2 specific inhibitors including NS-398 were found to attenuate Nrf2 nuclear accumulation in the acute phase of laminar shear stress [196]. Therefore, crosstalk between the Nrf2 and signaling of inflammatory mediators needs further investigation.

## 5. Incongruity in the Role of Nrf2 in Inflammasome Signaling

The pyrin domain containing 3 (NLRP3) inflammasome consists of an amino-terminal pyrin domain (PYD), a central NACHT domain and a carboxy-terminal leucine-rich repeat domain (LRR domain) [199]. NLRP3 acts as a sensor that detects a broad range of pathological signals including pathogen-associated molecular patterns (PAMPs), damage-associated molecular pattern molecules (DAMPs), cytosolic or mitochondrial ROS, microbial motifs, and many environmental signals [199]. Activated NLRP3 inflammasome, in turn, activates caspase 1, which results in the cleavage of pro- IL-1β and pro- IL-18 [200]. Caspases 4, 5 and 11 are also activated and subsequently cleave the substrate gasdermin D (GSDMD), which in turn inserts into the membrane thereby forming pores that ultimately trigger pyroptosis [201].

Multiple studies investigated potential mechanisms underlying the Nrf2-dependent regulation of NLRP3 inflammasome. Since Nrf2 is involved in the regulation of oxidative stress and antioxidant genes expression, it is believed to inhibit the activation of NLRP3 by suppressing ROS production [202]. Additionally, Nrf2 down-regulates the expression of genes involved in inflammasome assembly such as NLRP3, caspase 1, IL-1β, and IL-18, thereby inhibiting NLRP3 inflammasome activity [203]. Importantly, the known Nrf2 activator tert-butylhydroquinone (tBHQ), was found to induce the expression of NQO1 and to inhibit NLRP3 priming in Nrf2-ARE dependent manner [203].

These data suggest Nrf2 as a novel therapeutic target to inhibit the assembly of the NLRP3 inflammasome complex. In this context, several herbal extracts, and plant-derived compounds such as epigallocatechin-3-gallate and citral (3,7-dimethyl-2,6-octadienal) were found to suppress NLRP3 inflammasome activation in various animal models by engaging the Nrf2 signaling pathway [204,205,206]. For example, a flavonoid compound, isoliquiritigenin, was shown to inhibit ROS and activate Nrf2/ARE signaling which in turn suppressed the NLRP3 and NF-κB pathways [207]. Another natural polyphenol, mangiferin, inhibited several steps of the LPS/D-GalN induced NLRP3 inflammasome assembly complex including caspase-1, IL-1β, and TNF-α by promoting Nrf2 dependent HO-1 expression [208]. A very recent investigation showed that melatonin similarly was able to trigger Nrf2 signaling which in turn inhibited NLRP3 expression, ASC (apoptosis-associated speck-like protein) formation, caspase-1 cleavage, and Il-1β maturation and secretion [209]. In contrast; however, the activation of Nrf2 signaling by sulforaphane was shown to inhibit Nlrp3 inflammasome in bone marrow-derived macrophages (BMDMs) in Nrf2 independent manner [210]. While the negative modulation of inflammasome by sulforaphane was also confirmed in Alzheimer’s disease, the underlying mechanism appeared to be slightly different since this compound prevented A*β*1–42-induced caspase-1-dependent inflammasome activation, by suppressing STAT-1 phosphorylation, suggesting that multiple mechanisms might be involved in this pathway [211]. Similar results were also obtained in other studies testing the effects of sulforaphane and xanthohumol which essentially confirmed the inhibition of IL-1β by Nrf2/NQO1/HO-1 signaling pathway and GCL expression [212,213].

Importantly, there is contradictory evidence showing that Nrf2 activation is essential for the induction of NLRP3 and AIM2 inflammasome in LPS-primed BMDMs following different stimuli [214]. NLRP3 activators, such as silica, alum, cholesterol, and monosodium urate (MSU) crystals, failed to activate IL-1β secretion and the assembly of cytosolic ASC speck in Nrf2-deficient macrophages, suggesting that Nrf2 is essential for NLRP3 assembly under certain circumstances [214,215,216]. In addition to this, a role for Nrf2 mediated phagocytosis was also reported to play a role in the activation of NLRP3 and IL-β secretion in studies showing impaired phagocytosis in macrophages and high susceptibility to infections in Nrf2 knockout mice [217]. Consistent with this, another study reported the activation of NLRP3 inflammasome by silica crystals and aluminum salts via Nrf2 mediated phagocytosis [218].

However, it is still controversial whether activation of Nrf2 can lead to the prevention or the induction of aggressive inflammation in response to cholesterol or MSU treated macrophages. These conflicting results suggest the need to focus on the role of unknown functions of Nrf2 signaling pathway in hyperlipidemia, atherosclerosis, gout, and other inflammation-related diseases, which could enable the discovery of new therapeutics based on this pathway.

## 6. Nrf2 Dependent Anti-Inflammatory Drugs

Nonsteroidal anti-inflammatory drugs (NSAIDs) including traditional non-selective NSAIDS (nsNSAIDs) and cyclo-oxygenase-2 selective NSAIDs (COXIBs), are one of the most common medication for inflammation [219]. The primary activity of NSAIDs are enacted by blocking prostaglandins (PGs) synthesis through the cyclooxygenase enzymes (COX-1 and COX-2) inhibition. Aspirin is the most commonly used NSAID with antioxidant activity and reportedly activate Nrf2-ARE pathway and HO-1 expression in primary human melanocytes [220] as well as in neuronal apoptosis [221]. Similarly, celecoxib up-regulates heme oxygenase-1 (HO-1) expression in macrophages and vascular smooth muscle cells via redox signaling [222]. Treatment of celecoxib demonstrated increases in H-ferritin and TrxR1 mRNA levels respectively in Nrf2 dependent mechanism [223]. A widely used NSAID, diclofenac also activates Nrf2 expression and downstream related genes [224]. In choroidal neovascularization, indomethacin or bromfenac induces translocation of Nrf2 into the nucleus and up-regulates HO-1 expression [225]. On the other hand, pantoprazole, a proton pump inhibitor (PPIs) protected from NSAIDs-induced injury by inducing activation of Nrf2 through inactivation of Keap1 and by increasing expression of HO-1 in human gastric epithelial and endothelial cells and in animal model of gastric injury [226].

In vitro and in vivo studies on NO-NSAID (nitric oxide-donating NSAID) hybrid drugs such as NCX-4016 (mNO-ASA, nitric oxide-donating aspirin) and the isomeric NCX-4040, suggest significant potential for activation of Nrf2 through nitrosylation of Keap1 [227,228]. Moreover, cellular bioactivation of pNO-ASA yields a quinone methide, therefore, an alternative mechanism of Nrf2 activation by mNO-ASA is via an electrophilic quinone [229]. Analogues of NCX-4016 that are activated to quinone methide and without NO group, also showed covalent modification of Keap1, and induced Nrf2 translocation to the nucleus [230]. Accumulating evidence also suggests that treatment with small molecules such as 2-cyano-3,12-dioxooleana-1,9(11)-dien-28-oic acid- derivatives and sulforaphane induces Nrf2 activation and subsequently reduces significantly Th1 and Th17 cytokines including IL-6 by inhibiting recruitment of RNA polymerase II [231]. Similarly, pharmacological activation of mycophenolate mofetil, prevented the proinflammatory cytokines overexpression in a Nrf2 dependent mechanism [232]. In addition, short peptides such as casein glycomacropeptide hydrolysate were shown to have anti-inflammatory and antioxidant activity by increasing the Nrf2 nuclear translocation and HO-1 expression in RAW 264.7 macrophages and HepG2 cells [233,234]. Nrf2 induction by triazole derivatives inhibits pro- inflammatory cytokines release in different models of Huntington disease including primary mouse microglia, astrocytes, and *Drosophila* model and in cultured monocytes from human patients with huntington disease [235,236].

Several cell-derived metabolites such as itaconate and fumarate demonstrated anti-inflammatory responses in LPS- stimulated macrophages [237]. A cell-permeable derivative of itaconate, 4-octyl-itaconate (4-OI) demonstrated in vivo anti-inflammatory activity by induction of Nrf2 [237]. Here, the derivative of fumarate, dimethyl fumarate (DMF), is the only drug approved by US Food and Drug Administration and marketed by Biogen, as an anti-inflammatory therapeutic agent in multiple sclerosis with the ability to inhibit inflammation via Nrf2 antioxidant pathway [238,239]. In vivo studies in rodents had reported that DMF metabolite, monomethyl fumarate, activates Nrf2 by adduct formation at C151 in Keap1 in neuroinflammation [238]. Other commercial prodrugs of monomethyl fumarate such as diroximel fumarate is in a phase III clinical trial for multiple sclerosis and tepilamide fumarate, is in a phase II clinical trial for plaque psoriasis [240]. V ClinBio developed conjugate of monomethyl fumarate and eicosapentaenoic acid for simultaneous modulation of Nrf2 and NF-κB in cell lines and animal models of multiple sclerosis and psoriasis. Preclinical studies by Colby Pharmaceuticals developed a di- substituted hydroxylamine compound (OT551) which inhibits inflammation and oxidative stress by targeting Keap1 (OMEGA/NCT00485394). An ophthalmic solution of this compound protects from inflammation in retinal pigment epithelium and photoreceptors (OMEGA/NCT00485394).

## 7. Polyphenols and Nrf2 Signaling Can Modulate Inflammation

So far, we discussed different aspects of Nrf2 signaling pathway in inflammation, therefore, it is also worthwhile to discuss natural antioxidants which could modulate Nrf2-dependent treatment of inflammatory diseases [241]. In this context, dietary polyphenols are gaining interest and numerous studies supported their potential in animal and cell models. Polyphenols are secondary metabolites found in fruit and vegetables and are present either as glycosides esters or as free aglycones [241]. Several lines of evidence revealed that polyphenols could modulate oxidative stress and inflammation by inducing Nrf2 [242,243]. Curcumin, a polyphenol-derived compound, exhibits anti-inflammatory activity by Nrf2 activation and up-regulation of HO-1 expression [244]. A recent study demonstrated that curcumin inhibits the expression of both the inflammation mediators and of the matrix-degrading proteinases in inflammatory chondrocytes via ROS/Nrf2/HO-1-SOD2-NQO-1-GCLC signaling pathway [245]. In addition, bisdemethoxycurcumin, curcumin analog, induces activation of HO-1 expression through Ca^2+^/calmodulin-dependent protein kinase II–ERK1/2-Nrf2 cascade in LPS-stimulated macrophages [246]. Furthermore, demethoxy curcuminoids regulate Nrf2 mediated HO-1 expression via PI3K/Akt signaling pathway in *β*-cells [247]. Eriodictyol, a flavanone compound, exhibit anti-inflammatory and antioxidant activity by regulating Nrf2, inhibiting NF-ĸB, and inhibiting the expression of cytokines in macrophages [248,249]. Similarly, numerous phytochemicals such as resveratrol, lycopene, and luteolin exert prominent anti-inflammatory and antioxidant properties through the modulation of Nrf2 signaling pathway [250,251,252].

Epigallocatechin gallate (EGCG), another potent catechin antioxidant, ameliorates oxidative stress and inflammation by inducing both NF-κB and Nrf2 nuclear translocation and also promotes HO-1 expression in renal injury [253]. A similar trend of activation of Nrf2, ERK and p38 MAPK signaling pathways by EGCG was also observed against inflammation in different disease models such as oxalate-induced epithelial mesenchymal transition [254], obstructive nephropathy [253], fluoride induced lung inflammation [255] and diabetic nephropathy [256].

Flavonoids such as quercetin, naringenin and hesperidin-methyl-chalcone are well known for their anti-inflammatory properties. Hesperidin-methyl-chalcone inhibits oxidative stress and the release of inflammatory cytokines by inducing Nrf2 activation and HO-1 mRNA expression in gout induced inflammation in mice [257]. Similarly, naringenin promotes Nrf2/HO-1 signaling axis via PI_3_K/Akt signaling pathway in D-galactose-induced brain aging in mice [258]. Quercetin also demonstrated activation of Nrf2 and phase 2 enzymes HO-1, GPx1 and glutathione reductase (GR) in chronic arthritis [259,260]. Quercetin promotes Nrf2 activation by both Keap1-dependent and Keap1-independent mechanisms. Keap1-dependent mechanism involves the interaction of H-benzene and H-bond of quercetin to Keap-1 Arg415 and Tyr527, and Gly364, respectively. However, the Keap1-independent mechanism involves activation of JNK MAP kinase signaling pathway. Rosmarinic acid, a polyphenol-containing catechol, induces Nrf2 activation and attenuates chronic constriction injury (CCI)-induced neuropathic inflammation [261]. Rosmarinic acid induces Nrf2 activation through Akt/GSK-3β/Fyn pathway in β-amyloid-induced oxidative stress [262].

Kaurenoic acid, a diterpene, attenuated LPS-induced acute lung injury, neutrophil recruitment, and inflammatory cytokine gene expression by inducing Nrf2 activation and up-regulates the NQO-1, HO-1 and GCLC expressions [263]. Glycyrrhizin, a pentacyclic triterpenoid induces p38/Nrf2-dependent activation of HO-1 in LPS-induced Raw 264.7 cells [264].

## 8. Future Prospects

A correct approach for future research in the evaluation of the pharmacodynamic action of NRF2 inducers should assess a wide range of genetic and biochemical biomarkers (e.g., induction of the NQO1 transcript; epigenetic regulators of histone deacetylase, markers related to oxidative stress; pro and anti-inflammatory cytokines, NF-κB signaling molecules) in a dose-response manner by using validated and standardized methodologies. Additionally, in the preclinical studies, new disease directed animal models that best recapitulate human diseases concerning Nrf2-related pathogenic mechanisms should be developed to facilitate screening of new chemical entities as Nrf2 modulators. It is well known that non-communicable diseases are characterized by multiple pathways and complexity. The molecular mechanisms and histopathological pathways that lead to disease are different, but in many cases overlap. The research on a common molecular pathway such as the altered Nrf2 activity would help to facilitate the screening of new chemical entities as Nrf2 modulators or to consider re-proposing existing drugs. An area of potential interest for developing a candidate drug in the Nrf2 pathway would be the research on the indirect hallmarks of Nrf2 activation in diseased tissues that would represent a more robust and reliable readout of dosage regimen and drug distribution. More detailed studies are necessary to better characterize potential safety issues and to understand whether sustained Nrf2 induction could lead to the development of other diseases such as cancer. Furthermore, the functional characterization of the 27 cysteines retained by Keap1 and in particular, the identification of most reactive cysteines may also shed some light on the compounds which may modify Keap1 making it incapable of interacting with Nrf2 at both high as well as low-affinity sites. A major pitfall in the drugs acting on C151 is that they may interact with redox reactive cysteines in other proteins as well. Therefore, hierarchical modeling of target cysteines could have a potential advantage. Also, the studies of in vitro assays could identify off-target effects. Another concern which needs to be addressed is the inability of Nrf2 activators to cross the blood-brain barrier for therapeutic use in neurological disorders. Additionally, the therapeutic use of many Nrf2 inhibitors that have led to promising results in pre-clinical models still needs to be conclusively proven in rigorous clinical trials.

## 9. Conclusions

Nrf2-Keap1 signaling pathway is the hallmark of redox signaling and controlled inflammation and plays an important role in cell metabolic flexibility. Here, we reviewed ongoing scientific literature about regulation of Nrf2 signaling pathway in different aspects of inflammation such as cytokines, chemokine releasing factors, MMPs, and other inflammatory mediators affecting the NF-kB and MAPK networks to control inflammation. However, there are still some mechanisms such as interactions between Nrf2 and JAK/STAT signaling that needs to be investigated. Although many pharmaceutical companies are currently targeting Keap1, the prime regulator of Nrf2, it is still challenging to enhance the targeting of these compounds and their activity against these multifactorial complex diseases. Natural Nrf2 activators derived from plant sources as well as synthetic anti-inflammatory drugs require further experimental validation. Research for efficient therapeutic agents promoting Nrf2 activation came up with some new drugs which have entered clinical trials and will undoubtedly provide advancement in the management of inflammation in the near future. However, efforts are still ongoing to find new small molecule Nrf2 inducers with the advantages of oral administration, high target specificity, safe, and high bioavailability, therefore, present review would enable refinement in our understanding of Nrf2 signaling pathway interplay with expressions of associated target genes and support the hypothesis that Nrf2 inducers have the strong potential as anti-inflammatory therapeutic agents.
